# *rac*-Dimethyl 2-(*tert*-butyl­amino)-5-oxo-4,5-dihydro­pyrano[3,2-*c*]chromene-3,4-dicarboxyl­ate

**DOI:** 10.1107/S1600536811041493

**Published:** 2011-10-12

**Authors:** S. Antony Inglebert, K. Sethusankar, Yuvaraj Arun, Paramasivam T. Perumal

**Affiliations:** a Physics Department, Sri Ram Engineering College, Chennai 602 024, India; b Department of Physics, RKM Vivekananda College (Autonomous), Chennai 600 004, India; c Organic Chemistry Division, Central Leather Research Institute, Adyar, Chennai 600020, India

## Abstract

The title compound, C_20_H_21_NO_7_, is asymmetric with a chiral centre located in the pyran ring and crystallizes as a racemate. The mol­ecular framework is somewhat bent; the coumarin moiety and the pyran ring are inclined by 7.85 (5)°. The mol­ecular structure is characterized by an intra­molecular N—H⋯O hydrogen bond, which generates an *S*(6) ring motif, and the crystal packing is stabilized by inter­molecular C—H⋯O hydrogen bonds. The 3-carboxyl­ate O atom is involved in both of them, having a bifurcated character.

## Related literature

For the biological and pharmacological activity of coumarin and its derivatives, see: Borges *et al.* (2005 ▶); Gursoy & Karali (2003 ▶); Moffett (1964 ▶). For a related structure, see: Fun *et al.* (2011 ▶). For hydrogen-bond motifs, see: Bernstein *et al.* (1995 ▶).
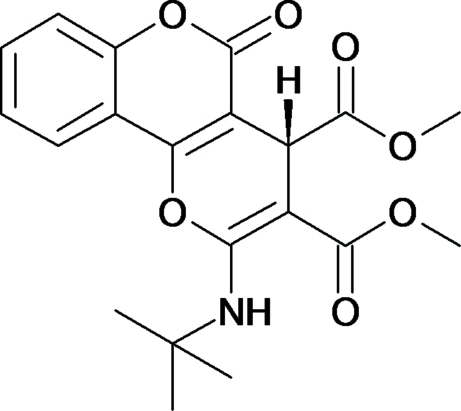


## Experimental

### 

#### Crystal data

C_20_H_21_NO_7_
*M* *_r_* = 387.38
Monoclinic, 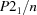

*a* = 10.0907 (2) Å
*b* = 16.3943 (4) Å
*c* = 11.8266 (2) Å
β = 107.941 (1)°
*V* = 1861.34 (7) Å^3^
*Z* = 4
Mo *K*α radiation
μ = 0.11 mm^−1^
*T* = 293 K
0.30 × 0.25 × 0.20 mm

#### Data collection

Bruker Kappa APEXII CCD diffractometer
Absorption correction: multi-scan (*SADABS*; Bruker, 2008 ▶) *T*_min_ = 0.969, *T*_max_ = 0.979
27295 measured reflections
6688 independent reflections
4069 reflections with *I* > 2σ(*I*)
*R*_int_ = 0.030

#### Refinement

*R*[*F* ^2^ > 2σ(*F* ^2^)] = 0.050
*wR*(*F* ^2^) = 0.158
*S* = 1.00
6688 reflections
258 parameters
H-atom parameters constrained
Δρ_max_ = 0.26 e Å^−3^
Δρ_min_ = −0.21 e Å^−3^

### 

Data collection: *APEX2* (Bruker, 2008 ▶); cell refinement: *SAINT* (Bruker, 2008 ▶); data reduction: *SAINT*; program(s) used to solve structure: *SHELXS97* (Sheldrick, 2008 ▶); program(s) used to refine structure: *SHELXL97* (Sheldrick, 2008 ▶); molecular graphics: *ORTEP-3* (Farrugia, 1997 ▶); software used to prepare material for publication: *SHELXL97* and *PLATON* (Spek, 2009 ▶).

## Supplementary Material

Crystal structure: contains datablock(s) global, I. DOI: 10.1107/S1600536811041493/ld2028sup1.cif

Structure factors: contains datablock(s) I. DOI: 10.1107/S1600536811041493/ld2028Isup2.hkl

Supplementary material file. DOI: 10.1107/S1600536811041493/ld2028Isup3.cml

Additional supplementary materials:  crystallographic information; 3D view; checkCIF report

## Figures and Tables

**Table 1:** Hydrogen-bond geometry (Å, °)

*D*—H⋯*A*	*D*—H	H⋯*A*	*D*⋯*A*	*D*—H⋯*A*
N1—H1⋯O5	0.86	1.97	2.6602 (16)	136
C19—H19*B*⋯O5^i^	0.96	2.49	3.4469 (19)	174

Symmetry code: (i) 
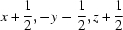
.

**Fig. 1.**
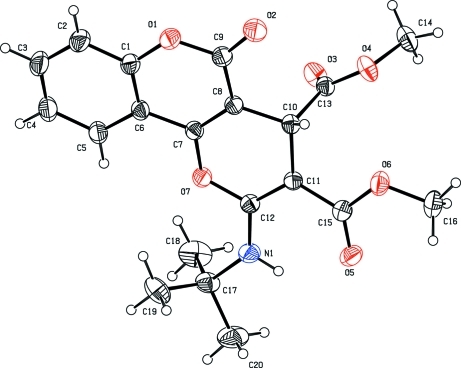
The molecular structure of the title compound with the atom numbering scheme. Displacement ellipsoids are drawn at 30% probability level. H atoms are presented as small spheres of arbitary radius.

**Fig. 2.**
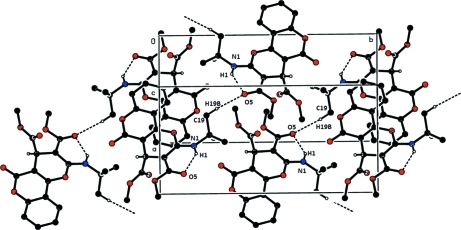
The packing arrangement of the title compound viewed down c axis, showing formation of hydrogen bond. Dashed lines indicate the intramolecular N—H···O and intermolecular C—H···O interactions.

## supplementary crystallographic information

### Comment

The coumarin and its derivatives, comprising many of the plant-derived compounds, display a wide range of biological activities such as antiviral, anti-inflammatory, anti-bacterial (Gursoy & Karali *et al.*, 2003), anti-fungal (Moffett *et al.* 1964), anticoagulant, and anti-proliferative. Some coumarin derivatives have been shown to be potential anti-HIV agents, antibiotics and antioxidants as well as flavour compounds(Borges *et al.*, 2005).

The title compound C_20_H_21_NO_7_ consists of a coumarin ring system fused with a pyran ring. The coumarin ring system is almost planar, with the C9 atom having a maximum deviation of only 0.0495 (15) Å. The coumarin ring system (O1/C1—C9) makes dihedral angles of 81.43 (7)° and 7.67 (5)° with the methyl carboxylates(C13/O3/O4/C14) and (C15/C16/O5/O6), respectively. The pyran ring forms dihedral angles of 88.77 (7)° and 2.95 (6)° with these two methyl carboxylates.

The X-ray crystal structure determination shows that the compound crystallizes as a racemate - the molecule has an asymmetric carbon atom C10. The title compound exhibits structural similarities with a previously reported related structure (Fun *et al.*, 2011).

The molecular structure is stabilized by an intramolecular N—H···O (Table 1) hydrogen bond which generates an S(6) ring motif (Bernstein *et al.*, 1995). The crystal packing is stabilized by intermolecular C—H···O hydrogen bonds. The carboxylate atom O5 is a bifurcated hydrogen acceptor - from the neighbouring *tert*-butyl group and from the amino group.

### Experimental

To a stirred solution of 4-hydroxy coumarin (0.162 g, 1.0 mmol) and dimethyl acetylenedicarboxylate (0.142 g, 1.0 mmol) in CH_3_CN (10 ml), a solution of *tert*-butyl isocynaide (0.083 g, 1.0 mmol) was added at room temperature over 5 min. The mixture was then stirred for 24 h. After completion of the reaction, the solvent was removed under vacuum and the solid residue was washed with n-hexane and re-crystallized from CH_2_Cl_2_/n-hexane(1:2) to give product as colourless crystals (0.337 g, 87%).

### Refinement

The positions of the hydrogen atoms bound to the N and C atoms were identified from the difference electron density maps and their distances were geometrically optimized. The hydrogen atoms bound to the C and N atoms were treated as riding atoms, with d(N—H)=0.86 and *U*iso(H) = 1.2Ueq(N) for amine group, d(C—H)=0.93 and *U*iso(H) = 1.2Ueq(C) for aromatic, d(C—H)=0.98 and *U*iso(H)=1.2Ueq(C) for methine and d(C—H)=0.96 and *U*iso(H) =1.5*U*eq(C) for methyl groups. Conformations of Me groups were rotationally optimized.

### Crystal data

**Table:**

C_20_H_21_NO_7_	*F*(000) = 816
*M**_r_* = 387.38	*D*_x_ = 1.382 Mg m^−^^3^
Monoclinic, *P*2_1_/*n*	Mo *K*α radiation, λ = 0.71073 Å
Hall symbol: -P 2yn	Cell parameters from 6688 reflections
*a* = 10.0907 (2) Å	θ = 2.2–32.5°
*b* = 16.3943 (4) Å	µ = 0.11 mm^−^^1^
*c* = 11.8266 (2) Å	*T* = 293 K
β = 107.941 (1)°	Block, colourless
*V* = 1861.34 (7) Å^3^	0.30 × 0.25 × 0.20 mm
*Z* = 4	

### Data collection

**Table:**

Bruker Kappa APEXII CCD diffractometer	6688 independent reflections
Radiation source: fine-focus sealed tube	4069 reflections with *I* > 2σ(*I*)
graphite	*R*_int_ = 0.030
ω and φ scans	θ_max_ = 32.5°, θ_min_ = 2.2°
Absorption correction: multi-scan (*SADABS*; Bruker, 2008)	*h* = −15→15
*T*_min_ = 0.969, *T*_max_ = 0.979	*k* = −24→24
27295 measured reflections	*l* = −17→17

### Refinement

**Table:**

Refinement on *F*^2^	Primary atom site location: structure-invariant direct methods
Least-squares matrix: full	Secondary atom site location: difference Fourier map
*R*[*F*^2^ > 2σ(*F*^2^)] = 0.050	Hydrogen site location: inferred from neighbouring sites
*wR*(*F*^2^) = 0.158	H-atom parameters constrained
*S* = 1.00	*w* = 1/[σ^2^(*F*_o_^2^) + (0.0737*P*)^2^ + 0.294*P*] where *P* = (*F*_o_^2^ + 2*F*_c_^2^)/3
6688 reflections	(Δ/σ)_max_ < 0.001
258 parameters	Δρ_max_ = 0.26 e Å^−^^3^
0 restraints	Δρ_min_ = −0.21 e Å^−^^3^

### Special details

**Table:**

Geometry. All e.s.d.'s (except the e.s.d. in the dihedral angle between two l.s. planes) are estimated using the full covariance matrix. The cell e.s.d.'s are taken into account individually in the estimation of e.s.d.'s in distances, angles and torsion angles; correlations between e.s.d.'s in cell parameters are only used when they are defined by crystal symmetry. An approximate (isotropic) treatment of cell e.s.d.'s is used for estimating e.s.d.'s involving l.s. planes.
Refinement. Refinement of *F*^2^ against ALL reflections. The weighted *R*-factor *wR* and goodness of fit *S* are based on *F*^2^, conventional *R*-factors *R* are based on *F*, with *F* set to zero for negative *F*^2^. The threshold expression of *F*^2^ > σ(*F*^2^) is used only for calculating *R*-factors(gt) *etc*. and is not relevant to the choice of reflections for refinement. *R*-factors based on *F*^2^ are statistically about twice as large as those based on *F*, and *R*- factors based on ALL data will be even larger.

### Fractional atomic coordinates and isotropic or equivalent isotropic displacement parameters (Å^2^)

**Table:**

	*x*	*y*	*z*	*U*_iso_*/*U*_eq_	
C1	0.60272 (14)	0.10657 (9)	0.40846 (13)	0.0459 (3)	
C2	0.68858 (16)	0.12542 (11)	0.52188 (14)	0.0574 (4)	
H2	0.7055	0.1794	0.5462	0.069*	
C3	0.74773 (16)	0.06267 (13)	0.59709 (15)	0.0632 (5)	
H3	0.8049	0.0745	0.6735	0.076*	
C4	0.72413 (16)	−0.01784 (12)	0.56175 (14)	0.0593 (4)	
H4	0.7647	−0.0595	0.6143	0.071*	
C5	0.64022 (15)	−0.03630 (10)	0.44824 (13)	0.0489 (3)	
H5	0.6253	−0.0904	0.4241	0.059*	
C6	0.57782 (13)	0.02628 (8)	0.36981 (12)	0.0405 (3)	
C7	0.48651 (12)	0.01446 (7)	0.25070 (11)	0.0371 (3)	
C8	0.42265 (13)	0.07696 (7)	0.18224 (11)	0.0373 (3)	
C9	0.44864 (15)	0.15958 (8)	0.22674 (13)	0.0458 (3)	
C10	0.32265 (12)	0.06565 (7)	0.06042 (11)	0.0364 (2)	
H10	0.3468	0.1037	0.0060	0.044*	
C11	0.33327 (13)	−0.02048 (7)	0.01863 (11)	0.0364 (3)	
C12	0.40362 (13)	−0.08012 (7)	0.09507 (11)	0.0376 (3)	
C13	0.17418 (13)	0.08403 (7)	0.06189 (12)	0.0385 (3)	
C14	−0.03365 (16)	0.15297 (11)	−0.03895 (17)	0.0615 (4)	
H14A	−0.0825	0.1019	−0.0530	0.092*	
H14B	−0.0695	0.1878	−0.1067	0.092*	
H14C	−0.0464	0.1786	0.0299	0.092*	
C15	0.26488 (14)	−0.04112 (8)	−0.10362 (12)	0.0416 (3)	
C16	0.11252 (17)	0.00960 (12)	−0.28374 (14)	0.0625 (4)	
H16A	0.1604	−0.0203	−0.3291	0.094*	
H16B	0.0811	0.0608	−0.3221	0.094*	
H16C	0.0339	−0.0214	−0.2785	0.094*	
C17	0.48307 (18)	−0.22850 (8)	0.13618 (14)	0.0519 (4)	
C18	0.4116 (3)	−0.24776 (12)	0.2283 (2)	0.0821 (6)	
H18A	0.4305	−0.2051	0.2867	0.123*	
H18B	0.4460	−0.2986	0.2662	0.123*	
H18C	0.3129	−0.2517	0.1904	0.123*	
C19	0.6380 (2)	−0.21543 (11)	0.19263 (19)	0.0730 (5)	
H19A	0.6809	−0.2064	0.1316	0.109*	
H19B	0.6783	−0.2628	0.2380	0.109*	
H19C	0.6532	−0.1688	0.2441	0.109*	
C20	0.4601 (3)	−0.29789 (10)	0.04631 (19)	0.0820 (6)	
H20A	0.3621	−0.3074	0.0117	0.123*	
H20B	0.5041	−0.3465	0.0857	0.123*	
H20C	0.4998	−0.2834	−0.0150	0.123*	
O1	0.54122 (11)	0.17100 (6)	0.33794 (10)	0.0537 (3)	
O2	0.39422 (14)	0.21939 (6)	0.17376 (11)	0.0651 (3)	
O3	0.12113 (11)	0.05152 (7)	0.12744 (10)	0.0568 (3)	
O4	0.11235 (10)	0.13887 (6)	−0.01951 (10)	0.0539 (3)	
O5	0.25835 (12)	−0.10881 (7)	−0.14909 (9)	0.0559 (3)	
O6	0.20492 (12)	0.02410 (6)	−0.16718 (9)	0.0545 (3)	
O7	0.47008 (10)	−0.06485 (5)	0.21316 (8)	0.0433 (2)	
N1	0.41676 (14)	−0.15701 (7)	0.06458 (11)	0.0503 (3)	
H1	0.3803	−0.1670	−0.0100	0.060*	

### Atomic displacement parameters (Å^2^)

**Table:**

	*U*^11^	*U*^22^	*U*^33^	*U*^12^	*U*^13^	*U*^23^
C1	0.0359 (6)	0.0521 (8)	0.0470 (7)	−0.0020 (5)	0.0088 (5)	−0.0071 (6)
C2	0.0439 (7)	0.0686 (10)	0.0542 (9)	−0.0058 (7)	0.0069 (7)	−0.0172 (8)
C3	0.0413 (7)	0.0934 (13)	0.0470 (8)	0.0027 (8)	0.0019 (6)	−0.0133 (9)
C4	0.0445 (8)	0.0835 (12)	0.0447 (8)	0.0112 (8)	0.0060 (6)	0.0050 (8)
C5	0.0423 (7)	0.0580 (8)	0.0429 (7)	0.0057 (6)	0.0080 (6)	0.0036 (6)
C6	0.0318 (5)	0.0473 (7)	0.0406 (7)	0.0012 (5)	0.0087 (5)	0.0000 (5)
C7	0.0345 (6)	0.0359 (6)	0.0396 (6)	−0.0013 (5)	0.0094 (5)	−0.0007 (5)
C8	0.0356 (6)	0.0333 (6)	0.0412 (6)	−0.0016 (5)	0.0090 (5)	−0.0004 (5)
C9	0.0456 (7)	0.0386 (6)	0.0497 (8)	−0.0015 (5)	0.0096 (6)	−0.0031 (6)
C10	0.0377 (6)	0.0314 (5)	0.0384 (6)	−0.0020 (4)	0.0092 (5)	0.0047 (5)
C11	0.0375 (6)	0.0330 (5)	0.0381 (6)	−0.0017 (4)	0.0108 (5)	0.0002 (5)
C12	0.0398 (6)	0.0333 (6)	0.0400 (6)	−0.0025 (5)	0.0127 (5)	0.0004 (5)
C13	0.0383 (6)	0.0323 (6)	0.0417 (7)	−0.0004 (5)	0.0074 (5)	0.0020 (5)
C14	0.0401 (7)	0.0598 (9)	0.0757 (11)	0.0082 (7)	0.0047 (7)	0.0113 (8)
C15	0.0404 (6)	0.0427 (7)	0.0416 (7)	−0.0055 (5)	0.0126 (5)	0.0008 (5)
C16	0.0517 (8)	0.0845 (12)	0.0422 (8)	−0.0023 (8)	0.0013 (6)	0.0040 (8)
C17	0.0706 (9)	0.0321 (6)	0.0569 (9)	0.0052 (6)	0.0255 (7)	0.0088 (6)
C18	0.1131 (17)	0.0600 (10)	0.0932 (15)	0.0014 (11)	0.0612 (14)	0.0179 (10)
C19	0.0713 (11)	0.0536 (9)	0.0914 (14)	0.0189 (8)	0.0212 (10)	0.0255 (9)
C20	0.1286 (19)	0.0364 (8)	0.0855 (14)	0.0102 (10)	0.0396 (13)	−0.0010 (8)
O1	0.0546 (6)	0.0424 (5)	0.0553 (6)	−0.0035 (4)	0.0039 (5)	−0.0106 (5)
O2	0.0781 (8)	0.0363 (5)	0.0692 (8)	0.0051 (5)	0.0054 (6)	−0.0007 (5)
O3	0.0503 (6)	0.0632 (7)	0.0625 (7)	0.0087 (5)	0.0255 (5)	0.0193 (5)
O4	0.0411 (5)	0.0509 (6)	0.0659 (7)	0.0077 (4)	0.0108 (5)	0.0225 (5)
O5	0.0677 (7)	0.0489 (6)	0.0474 (6)	−0.0022 (5)	0.0121 (5)	−0.0104 (5)
O6	0.0598 (6)	0.0511 (6)	0.0417 (5)	−0.0015 (5)	−0.0002 (5)	0.0038 (4)
O7	0.0514 (5)	0.0332 (4)	0.0405 (5)	0.0023 (4)	0.0074 (4)	0.0026 (4)
N1	0.0667 (8)	0.0336 (5)	0.0473 (7)	0.0054 (5)	0.0127 (6)	−0.0004 (5)

### Geometric parameters (Å, °)

**Table:**

C1—O1	1.3703 (18)	C13—O4	1.3245 (15)
C1—C2	1.389 (2)	C14—O4	1.4380 (18)
C1—C6	1.3905 (19)	C14—H14A	0.9600
C2—C3	1.371 (3)	C14—H14B	0.9600
C2—H2	0.9300	C14—H14C	0.9600
C3—C4	1.383 (3)	C15—O5	1.2261 (16)
C3—H3	0.9300	C15—O6	1.3395 (17)
C4—C5	1.382 (2)	C16—O6	1.4262 (18)
C4—H4	0.9300	C16—H16A	0.9600
C5—C6	1.3962 (19)	C16—H16B	0.9600
C5—H5	0.9300	C16—H16C	0.9600
C6—C7	1.4392 (18)	C17—N1	1.4797 (18)
C7—C8	1.3415 (17)	C17—C18	1.514 (2)
C7—O7	1.3674 (15)	C17—C19	1.514 (3)
C8—C9	1.4474 (18)	C17—C20	1.525 (2)
C8—C10	1.4942 (17)	C18—H18A	0.9600
C9—O2	1.2016 (17)	C18—H18B	0.9600
C9—O1	1.3714 (18)	C18—H18C	0.9600
C10—C11	1.5107 (17)	C19—H19A	0.9600
C10—C13	1.5335 (17)	C19—H19B	0.9600
C10—H10	0.9800	C19—H19C	0.9600
C11—C12	1.3719 (17)	C20—H20A	0.9600
C11—C15	1.4375 (18)	C20—H20B	0.9600
C12—N1	1.3288 (16)	C20—H20C	0.9600
C12—O7	1.3733 (16)	N1—H1	0.8600
C13—O3	1.1948 (16)		
			
O1—C1—C2	116.55 (14)	H14A—C14—H14B	109.5
O1—C1—C6	121.84 (12)	O4—C14—H14C	109.5
C2—C1—C6	121.59 (14)	H14A—C14—H14C	109.5
C3—C2—C1	118.49 (16)	H14B—C14—H14C	109.5
C3—C2—H2	120.8	O5—C15—O6	121.50 (13)
C1—C2—H2	120.8	O5—C15—C11	126.77 (13)
C2—C3—C4	121.35 (15)	O6—C15—C11	111.74 (11)
C2—C3—H3	119.3	O6—C16—H16A	109.5
C4—C3—H3	119.3	O6—C16—H16B	109.5
C5—C4—C3	119.97 (16)	H16A—C16—H16B	109.5
C5—C4—H4	120.0	O6—C16—H16C	109.5
C3—C4—H4	120.0	H16A—C16—H16C	109.5
C4—C5—C6	120.02 (15)	H16B—C16—H16C	109.5
C4—C5—H5	120.0	N1—C17—C18	110.21 (14)
C6—C5—H5	120.0	N1—C17—C19	111.32 (12)
C1—C6—C5	118.57 (13)	C18—C17—C19	111.62 (16)
C1—C6—C7	116.46 (12)	N1—C17—C20	104.32 (13)
C5—C6—C7	124.96 (13)	C18—C17—C20	110.02 (15)
C8—C7—O7	122.87 (11)	C19—C17—C20	109.10 (16)
C8—C7—C6	122.11 (12)	C17—C18—H18A	109.5
O7—C7—C6	115.02 (11)	C17—C18—H18B	109.5
C7—C8—C9	119.72 (12)	H18A—C18—H18B	109.5
C7—C8—C10	122.91 (11)	C17—C18—H18C	109.5
C9—C8—C10	117.36 (11)	H18A—C18—H18C	109.5
O2—C9—O1	117.10 (12)	H18B—C18—H18C	109.5
O2—C9—C8	124.86 (13)	C17—C19—H19A	109.5
O1—C9—C8	118.04 (12)	C17—C19—H19B	109.5
C8—C10—C11	109.67 (10)	H19A—C19—H19B	109.5
C8—C10—C13	109.67 (10)	C17—C19—H19C	109.5
C11—C10—C13	110.81 (10)	H19A—C19—H19C	109.5
C8—C10—H10	108.9	H19B—C19—H19C	109.5
C11—C10—H10	108.9	C17—C20—H20A	109.5
C13—C10—H10	108.9	C17—C20—H20B	109.5
C12—C11—C15	119.05 (11)	H20A—C20—H20B	109.5
C12—C11—C10	121.68 (11)	C17—C20—H20C	109.5
C15—C11—C10	119.25 (11)	H20A—C20—H20C	109.5
N1—C12—C11	124.89 (12)	H20B—C20—H20C	109.5
N1—C12—O7	112.99 (11)	C1—O1—C9	121.69 (11)
C11—C12—O7	122.12 (11)	C13—O4—C14	117.09 (12)
O3—C13—O4	124.71 (12)	C15—O6—C16	117.20 (12)
O3—C13—C10	123.67 (11)	C7—O7—C12	118.42 (10)
O4—C13—C10	111.61 (11)	C12—N1—C17	131.41 (13)
O4—C14—H14A	109.5	C12—N1—H1	114.3
O4—C14—H14B	109.5	C17—N1—H1	114.3
			
O1—C1—C2—C3	177.39 (13)	C13—C10—C11—C15	69.62 (14)
C6—C1—C2—C3	−1.0 (2)	C15—C11—C12—N1	1.5 (2)
C1—C2—C3—C4	0.4 (2)	C10—C11—C12—N1	179.80 (12)
C2—C3—C4—C5	0.5 (3)	C15—C11—C12—O7	−179.35 (11)
C3—C4—C5—C6	−0.8 (2)	C10—C11—C12—O7	−1.03 (18)
O1—C1—C6—C5	−177.67 (12)	C8—C10—C13—O3	−54.74 (16)
C2—C1—C6—C5	0.6 (2)	C11—C10—C13—O3	66.48 (17)
O1—C1—C6—C7	1.41 (19)	C8—C10—C13—O4	126.41 (12)
C2—C1—C6—C7	179.72 (13)	C11—C10—C13—O4	−112.38 (12)
C4—C5—C6—C1	0.3 (2)	C12—C11—C15—O5	2.2 (2)
C4—C5—C6—C7	−178.70 (13)	C10—C11—C15—O5	−176.18 (13)
C1—C6—C7—C8	−3.39 (18)	C12—C11—C15—O6	−178.03 (11)
C5—C6—C7—C8	175.63 (13)	C10—C11—C15—O6	3.61 (16)
C1—C6—C7—O7	176.64 (11)	C2—C1—O1—C9	−176.45 (13)
C5—C6—C7—O7	−4.34 (18)	C6—C1—O1—C9	1.9 (2)
O7—C7—C8—C9	−178.01 (11)	O2—C9—O1—C1	176.14 (13)
C6—C7—C8—C9	2.02 (19)	C8—C9—O1—C1	−3.3 (2)
O7—C7—C8—C10	2.75 (19)	O3—C13—O4—C14	−7.5 (2)
C6—C7—C8—C10	−177.22 (11)	C10—C13—O4—C14	171.38 (12)
C7—C8—C9—O2	−178.08 (14)	O5—C15—O6—C16	11.0 (2)
C10—C8—C9—O2	1.2 (2)	C11—C15—O6—C16	−168.79 (12)
C7—C8—C9—O1	1.36 (19)	C8—C7—O7—C12	10.56 (18)
C10—C8—C9—O1	−179.36 (11)	C6—C7—O7—C12	−169.47 (10)
C7—C8—C10—C11	−13.49 (16)	N1—C12—O7—C7	167.98 (11)
C9—C8—C10—C11	167.26 (11)	C11—C12—O7—C7	−11.28 (17)
C7—C8—C10—C13	108.41 (13)	C11—C12—N1—C17	−177.04 (14)
C9—C8—C10—C13	−70.84 (14)	O7—C12—N1—C17	3.7 (2)
C8—C10—C11—C12	12.52 (16)	C18—C17—N1—C12	61.5 (2)
C13—C10—C11—C12	−108.70 (13)	C19—C17—N1—C12	−62.9 (2)
C8—C10—C11—C15	−169.16 (10)	C20—C17—N1—C12	179.60 (16)

### Hydrogen-bond geometry (Å, °)

**Table:**

*D*—H···*A*	*D*—H	H···*A*	*D*···*A*	*D*—H···*A*
N1—H1···O5	0.86	1.97	2.6602 (16)	136
C19—H19B···O5^i^	0.96	2.49	3.4469 (19)	174.

Symmetry codes: (i) *x*+1/2, −*y*−1/2, *z*+1/2.
